# Emulating Non-Hermitian Dynamics in a Finite Non-Dissipative Quantum System

**DOI:** 10.3390/e25091256

**Published:** 2023-08-24

**Authors:** Eloi Flament, François Impens, David Guéry-Odelin

**Affiliations:** 1 Laboratoire Collisions, Agrégats, Réactivité, FeRMI, Université de Toulouse, CNRS, UPS, 118 Route de Narbonne, 31062 Toulouse, France; eloi.flament@univ-tlse3.fr; 2 Instituto de Física, Universidade Federal do Rio de Janeiro, Rio de Janeiro 21941-972, RJ, Brazil; impens@if.ufrj.br

**Keywords:** open quantum system, quantum simulators, non-Hermitian systems, non-Markovian dynamics

## Abstract

We discuss the emulation of non-Hermitian dynamics during a given time window using a low-dimensional quantum system coupled to a finite set of equidistant discrete states acting as an effective continuum. We first emulate the decay of an unstable state and map the quasi-continuum parameters, enabling the precise approximation of non-Hermitian dynamics. The limitations of this model, including in particular short- and long-time deviations, are extensively discussed. We then consider a driven two-level system and establish criteria for non-Hermitian dynamics emulation with a finite quasi-continuum. We quantitatively analyze the signatures of the finiteness of the effective continuum, addressing the possible emergence of non-Markovian behavior during the time interval considered. Finally, we investigate the emulation of dissipative dynamics using a finite quasi-continuum with a tailored density of states. We show through the example of a two-level system that such a continuum can reproduce non-Hermitian dynamics more efficiently than the usual equidistant quasi-continuum model.

## 1. Introduction

The decay of unstable states occurs in a wide range of areas of quantum mechanics, including atomic physics, with the limited lifetime of excited electronic states in atoms; condensed matter with various relaxation processes in quantum dot electronic states; in polaron and exciton physics; nuclear physics, with the exponential decay law in radioactivity; and high-energy physics, with the short lifetime of particles such as the Higgs boson. The basic phenomenon underlying these decays is fundamentally the same. It is the irreversible transition from an initial unstable state to a continuum of final states. Such a decay can be derived from first principles. Within the perturbative limit, this problem often offers a first introduction to open quantum systems with Fermi’s golden rule. Besides the perturbative limit, the complete resolution of the model reveals three different successive regimes characterized by different decay laws [1,2,3]: with very short time [4], the decay is quadratic, it is subsequently governed by an exponential law at intermediate time, and eventually exhibits a power law tail at long time scales [5,6]. In general, these studies reveal that a decay can be sensitive to the structure of the environment.

Quantum simulations have become a very important research topic, with various fundamental and technological applications [7]. As any realistic quantum process involves a finite amount of dissipation, a quantum decay emulator appears as an interesting building block for such systems. The simplest model of quantum decay corresponds to the inclusion of a non-Hermitian contribution to the Hamiltonian, which allows emulating non-Hermitian systems. Non-Hermitian dynamics also have their own interest. Since the realization of complex optical PT potentials [8,9], the community has unveiled a very rich phenomenology and numerous applications for effective non-Hermitian systems. To name a few, we can mention the non-Hermitian skin effect [10,11], non-Hermitian transport [8,12,13,14], and more generally the intriguing topology of effective non-Hermitian systems [15,16,17,18,19,20,21]. Emulating non-Hermitian dynamics can provide access to the above phenomena using different platforms.

Engineering truly non-Hermitian and irreversible quantum dynamics over an arbitrarily long time requires the interaction of the system with an infinite set of states, as in the usual paradigm of infinite discrete quasi-continuum [22,23]. Nevertheless, the emulation of quantum dissipation during a finite time can be sufficient for experimental purposes; for instance, when dissipation is used as an asset to prepare a given quantum state [24]. In this context, simulating dissipative quantum dynamics thanks to coupling with a finite—and ideally minimal—number of ancilla states seems a feasible task. This possibility may have interesting applications in quantum computing, where a smaller number of ancilla states usually corresponds to a simpler setup.

**Figure 1:**
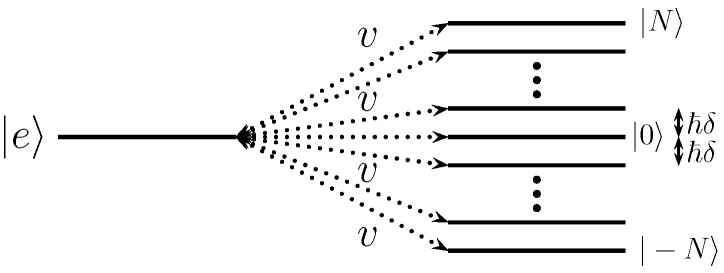
Schematic picture of a quantum system (consisting here of a single discrete state |e〉) coupled to a finite set of equidistant discrete levels. This model mimics the coupling to a continuum.

The purpose of this article is to investigate this avenue and provide an emulation of non-Hermitian dynamics for a given time interval with a quasi-continuum made of a *finite* set of ancilla states (see Figure 1). We use the trace distance to quantify the quality of our model, and discuss in detail the minimum number of levels required to obtain an accurate emulation. We also investigate separately the short- and long-term behavior of the associated dynamics. At early times, we compare the quantum evolution of the coupled system with the Zeno effect expected from a genuine continuum. At long times, we observe and characterize quantitatively the emergence of revivals in the presence of the finite continuum, enabling us to set an upper limit for the validity time of this emulation. We connect the appearance of these revivals with adequate measures of non-Markovianity.

We proceed as follows: In Section 2, we provide a brief reminder of the decay for a single discrete level coupled to an infinite continuum. Section 3 presents the considered quasi-continuum model, composed of equidistant energy levels equally coupled to a given state, and discuss its main features. In Section 4, we investigate the same issues for a two-level system whose excited state is coupled to a continuum. We identify a method for defining the minimum size of the discrete continuum using Fourier analysis. In Section 5, we discuss the emergence of non-Makovian evolution at long times and build on the previous sections to design a discrete quasi-continuum with the minimum number of states to reproduce the expected behavior in the strong coupling limit.

## 2. Decay of a Single Level Coupled to an Effective Continuum

We illustrate our method by first considering a system consisting of a single eigenstate |e〉 coupled to a large set of independent states $\left\{|\varphi_{f}\rangle \right\}$. This system is the usual paradigm explaining the irreversible exponential decay and Lamb shift undergone by a quantum state coupled to a continuum [23]. We briefly recall below the corresponding derivation in the standard case of an infinite and broad effective continuum consisting of the set of states $\left\{|\varphi_{f}\rangle \right\}$. The quantum system under consideration follows a Hamiltonian given by the sum $H=H_{0}+V$ with $H_{0}=E_{e}\left|e\rangle \langle e\right|+\sum_{f}E_{f}|\varphi_{f}\rangle \langle \varphi_{f}|$ the free-system Hamiltonian diagonal in the basis $\{|e\rangle ,|\varphi_{f}\rangle \}$, and with the off-diagonal contribution $V=\sum_{f}V_{fe}|\varphi_{f}\rangle \langle e|+h.c.$ accounting for the coupling between the discrete state and the effective environment.

We search for a solution to a time-dependent Schrödinger of the form:

(1) $$|\psi \left(t\right)\rangle =c_{e}\left(t\right)e^{-iE_{e}t/\hbar }|e\rangle +\sum_{f}c_{f}\left(t\right)e^{-iE_{f}t/\hbar }|\varphi_{f}\rangle .$$

and subsequently obtain by projection on the eigenstates of $H_{0}$ the following integro-differential obeying the coefficient $c_{e}$:

(2) $${\dot{c}}_{e}\left(t\right)=-\int_{0}^{t}dt^{\prime }K\left(t-t^{\prime }\right)c_{e}\left(t^{\prime }\right),$$

where the kernel is defined by

(3) $$K\left(\tau \right)=\frac{1}{\hbar^{2}}\sum_{f}{\left|V_{ef}\right|}^{2}e^{i\omega_{ef}\tau },$$

with $\omega_{ef}=\left(E_{e}-E_{f}\right)/\hbar$. Equations (2) and (3) capture the exact quantum dynamics of this system and so far involve no assumptions about the set of final states $\left\{|\varphi_{f}\rangle \right\}$. The function K(τ) accounts for the memory of the effective environment, resulting in a possibly non-Markovian evolution for the amplitude $c_{e}\left(t\right)$.

We now assume that the effective continuum $\left\{|\varphi_{f}\rangle \right\}$ covers a wide range of frequencies. As a result, the K(τ) function is expected to peak sharply around τ=0 when compared to the time-scale of the amplitude evolution; for a genuine continuum with a flat coupling, the sum over all possible final states in Equation (3) would actually yield a Dirac-like distribution. This large timescale separation enables one to pull out the amplitude $c_{e}\left(t\right)$ from the integration of the memory kernel in Equation (2) and to extend the boundary of this integral to infinity. We then obtain a simple closed differential equation for $c_{e}$:

(4) $${\dot{c}}_{e}\left(t\right)=-\left(\int_{0}^{\infty }d\tau K\left(\tau \right)\right)c_{e}\left(t\right).$$

The pre-factor is readily derived within the framework of complex analysis:

(5) $$\int_{0}^{\infty }d\tau K\left(\tau \right)=i\Delta \omega_{e}+\frac{\Gamma }{2},$$

with

(6) $$\begin{array}{ccc} \frac{\Gamma }{2}=\frac{2\pi }{\hbar }\sum_{f}{\left|V_{ef}\right|}^{2}\delta \left(E_{e}-E_{f}\right),\;\;\mathrm{and}\;\;\Delta \omega_{e} & = & \frac{1}{\hbar }P\left(\sum_{f}\frac{|V_{ef}{|}^{2}}{E_{e}-E_{f}}\right), \end{array}$$

where P denotes the principal value. For the considered coupling to a large set of states, the main effects on the discrete state are therefore an exponential decay of the population at a rate Γ witnessing an irreversible evolution as well as a frequency shift $\Delta \omega_{e}$, commonly referred to as the Lamb shift. Equation (6) simply expresses Fermi’s golden rule for the effective continuum with the density of states $\rho \left(E\right)=\sum_{f}\delta \left(E-E_{f}\right)$. Remarkably, Fermi’s golden rule holds, not only for a genuine continuum, but also for a countable set $\left\{|\varphi_{f}\rangle \right\}$ involving only discrete states [23]. Finally, unlike Equation (2), the amplitude $c_{e}\left(t\right)$ at a given time no longer depends on its history; the effective continuum $\left\{|\varphi_{f}\rangle \right\}$ behaves as a Markovian environment. Equations (4) and (5) implicitly define an effective non-Hermitian Hamiltonian $H_{\mathrm{eff}}=\Delta \omega_{e}-i\frac{\Gamma }{2}$ for this one-level system.

A closer look at Equation (6) reveals the central role played by the density of states ρ(E) of the effective continuum [3,25,26,27]. Indeed, its properties are responsible for deviations to the exponential law both at short and long times; the existence of an energy threshold ($\rho (E<E_{0})=0$) generates long-time deviations, while the finiteness of the mean energy (∫ρ(E)EdE<∞) explains the short time deviations.

In the same spirit, we examine below how the two characteristics of the quantum evolution discussed above—exponential decay and non-Markovianity—are affected by the use of a finite set as an effective continuum. We restrict our attention to a finite time-interval, as only infinite sets can reproduce these characteristics during arbitrary long times.

## 3. Coupling of a Single State to a Finite Discretized Continuum

*Description of the FQC model*. To quantitatively characterize such an irreversible process, we introduce a finite quasi-continuum (FQC) model consisting of a finite set of equidistant energy levels, which are equally coupled to a given state |e〉 (See Figure 1). This system mimics the decay of an unstable discrete state |e〉 in a finite time window. In what follows, unless otherwise stated, we always consider FQCs composed of $N_{\mathrm{FQC}}=2N+1$ equidistant energy levels symmetrically distributed around the unstable state energy, set by convention to E=0. Here, the total Hilbert space is of dimension $N_{\mathrm{tot}}=N_{\mathrm{sys}}+N_{\mathrm{FQC}}=2N+2$. We denote with ℏδ the energy gap between two successive FQC states and with $v=|V_{fe}|$ the flat coupling strength between the FQC and the discrete state |e〉. The expected decay Γ in the limit N→+∞ is given by Equation (6), which captures the dynamics of an infinite discrete continuum, namely

(7) $$\Gamma =\frac{2\pi }{\hbar^{2}}\frac{v^{2}}{\delta }.$$

which corresponds to Fermi’s golden rule. In the following, we consider FQCs associated with a fixed common decay rate Γ. We therefore impose $v^{2}/\delta =Cte$. In our numerical resolution, we implicitly normalize the energies using ℏΓ and the time using $\Gamma^{-1}$, which amounts to taking ℏ=1 and Γ=1. Our results are valid for arbitrary values of the dissipation rate Γ as long as the dimensionless parameters $\bar{v}=v/\left(\hbar \Gamma \right)$, ${\bar{t}}_{f}=\Gamma t_{f},$… remain identical. The considered FQCs are therefore entirely determined by their size (2N+1) and the coupling strength, *v*.

The model Hamiltonian in matrix form reads

(8) $$H=\left(\begin{array}{ccccccc} 0 & v & ... & v & v & v & v\\ v & -N\hbar \delta & 0 & ... & 0 & 0 & 0\\ v & 0 & -(N-1)\hbar \delta & 0 & ... & 0 & 0\\ v & 0 & ... & 0 & ... & 0 & 0\\ v & 0 & . & . & 0 & (N-1)\hbar \delta & 0\\ v & 0 & 0 & 0 & ... & 0 & N\hbar \delta \end{array}\right).$$

**Figure 2:**
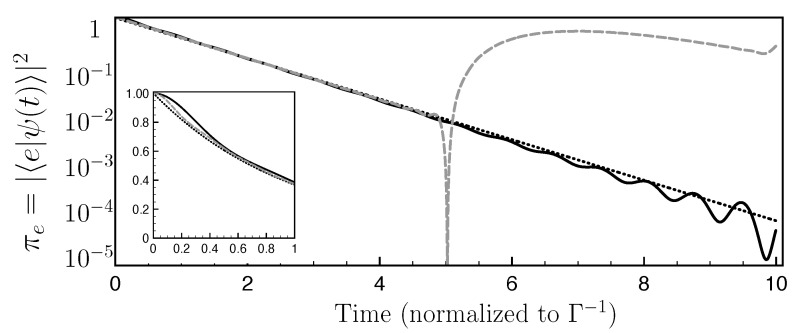
FQC vs. genuine continuum for a single discrete level: Excited state population $\pi_{e}\left(t\right)$ of a discrete level coupled to FQCs obtained from the full quantum dynamics under the Hamiltonian (8) with N=15 and v=0.3ℏΓ (solid black line) and N=15 and v=0.45ℏΓ (gray dashed line) as a function of time (normalized to $\Gamma^{-1}$). The dotted line represents the exponential decay expected from a genuine continuum.

*Examples of FQCs and connection with the Zeno effect.* In Figure 2, we compare the evolution of the excited state population for an example of FQC (solid black line) with the exponential decay expected from Fermi’s golden rule (dotted line). As expected, we observe a very good agreement, with minor discrepancies at short times (see the inset of Figure 2) and at long times when the population is extremely small. We used a FQC with N=15 and a coupling strength v=0.3ℏΓ. In this case, the emulation of quantum decay does not require a very large Hilbert space.

The disagreement at short times corresponds to a quadratic decay of the excited state coupled to a FQC. The initial quadratic profile is directly related to the Zeno effect. This is found by expanding the evolution operator for a short amount of time δt, by writing $|\psi \left(\delta t\right)\rangle =e^{-iH\delta t/\hbar }|e\rangle ≃|e\rangle +|\delta \psi \rangle$ with

(9) $$|\delta \psi \rangle =\left(-\frac{iH}{\hbar }\delta t-\frac{H^{2}}{2\hbar^{2}}{\left(\delta t\right)}^{2}\right)|e\rangle .$$

We infer the initial state population $\pi_{e}\left(t\right)={\left|\langle e|\psi \left(t\right)\rangle \right|}^{2}$ at early times

(10) $$\pi_{e}\left(\delta t\right)≃1-\frac{{\left(\delta t\right)}^{2}}{T_{Z}^{2}},$$

where $T_{Z}^{-2}=\frac{1}{\hbar^{2}}\left({\langle H^{2}\rangle }_{e}-{\langle H\rangle }_{e}^{2}\right)=\frac{1}{\hbar^{2}}\sum_{n}\langle e|V|n\rangle \langle n|V|e\rangle =\left(2N+1\right){\left(v/\hbar \right)}^{2}$. The duration $T_{Z}$ corresponds to the Zeno time and decreases with the size of the FQC. As $T_{Z}$ vanishes in the limit N→+∞, the observed initial quadratic profile witnesses the limited number of states of the FQC. For the parameters N=15 and v=0.3ℏΓ, one finds $T_{Z}≃0.6\;\Gamma^{-1}$, consistent with the inset in Figure 2.

We now provide a second example of FQC, for which the excited state population evolves very differently from the expected exponential decay. We take a FQC with N=15 and v=0.45ℏΓ, which corresponds to a larger energy gap between the FQC levels than in the first example, therefore being further away from an ideal continuum. Good agreement is observed up to $t≃5\Gamma^{-1}$, when the population $\pi_{e}\left(t\right)$ grows abruptly (gray dashed line, Figure 2). This revival of the probability distribution in the discrete state reveals the underlying fully coherent dynamics.

**Figure 3:**
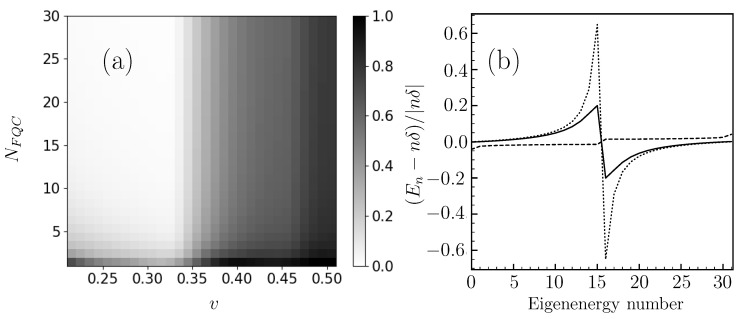
(**a**) Quality of the emulation of a continuum using a FQC: Parameter $D_{1}$ (obtained by a numerical resolution of the Shcrödinger equation) as a function of the FQC parameters {N,v} (*v* is given in units of ℏΓ). The white zone reveals a very good agreement with the exponential decay expected from a genuine continuum. (**b**) Spectrum analysis of the Hamiltonian (8): Eigenenergies $E_{n}$ in crescent order normalized by δ for N=15 and δ=5Γ (dotted line), δ=0.5Γ (solid line) and δ=0.05Γ (dashed line). $N_{FQC}=2N+1$ is the FQC size.

*Quantitative mapping of successful FQCs for the emulation of a single-state decay*. We now proceed to a quantitative mapping of the FQC parameters (N,v) suitable for accurate continuum emulation. In order to capture the accuracy of our model for a given time window, one needs a distance measure between the quantum evolution observed in the presence of an FQC and the genuine continuum. For the single-state quantum system considered here, the density matrix boils down to the excited state population $\pi_{e}\left(t\right)$. We therefore introduce the following distance

(11) $$D_{1}\left(t_{f}\right)=\frac{1}{t_{f}}\int_{0}^{t_{f}}\left|\pi_{e}\left(t\right)-\pi_{0}\left(t\right)\right|dt.$$

as a figure of merit for the quality of the FQC emulation over the time window $0\leq t\leq t_{f}$. $\pi_{0}\left(t\right)=\pi_{e}\left(0\right)e^{-\Gamma t}$ is the exponential decay expected in the large continuum limit. We choose $t_{f}$ to be larger than several $\Gamma^{-1}$ to best account for the full decay. In our numerical examples, we systematically use $t_{f}=10\Gamma^{-1}$ (unless otherwise specified). The results are summarized in Figure 3a. The good set of parameters for the chosen time interval is provided by the white area. This figure reveals that the quality of the emulation increases with the number of FQC states and decreases with the potential strength *v*, corresponding to FQCs with a larger energy gap ℏδ for a fixed decay rate Γ. In particular, the quality of the emulation drops off sharply above a critical coupling value $v_{c}≃0.32\;\hbar \Gamma$, which is independent of the number of FQC states. We explain below this abrupt change in terms of quantum interference and revivals of the discrete state population. The dashed gray line of Figure 2 provides an example of the revival of the excited population $\pi_{e}\left(t\right)$ coupled to a FQC with a strength $v\geq v_{c}$.

We now provide a quantitative analysis of the occurrence of such revivals in a given time window. We first look for a necessary condition of revival. For this purpose, we expand the wave function at time *t* on the eigenbasis:

(12) $$|\psi \left(t\right)\rangle =\sum_{n=0}^{N_{\mathrm{tot}}}a_{n}\left(0\right)e^{-iE_{n}t/\hbar }|\psi_{n}\rangle ,$$

where $E_{n}$ are the eigenenergies of the total Hamiltonian (8) and with $N_{\mathrm{tot}}=2N+2$ the dimension of the total Hilbert space. We denote by $T_{r}$ the revival time, which necessarily fulfills

(13) $$|||\psi \left(T_{r}\right)\rangle -|\psi \left(0\right){\rangle ||}^{2}=2\sum_{n=0}^{N_{\mathrm{tot}}}{\left|a_{n}\left(0\right)\right|}^{2}\left(1-\cos\left(E_{n}T_{r}/\hbar \right)\right)\equiv \varepsilon \ll 1.$$

**Figure 4:**
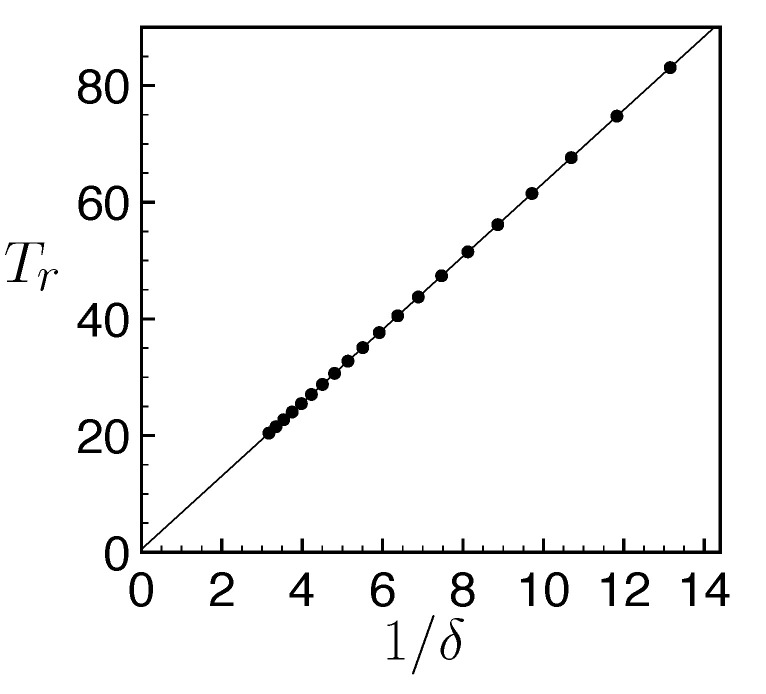
Black disks: Resurgence time $T_{r}$ as a function of the inverse of the FQC energy gap ℏδ. $T_{r}$ is obtained using a numerical resolution of the Schrödinger equation with the Hamiltonian (8). The solid black line represents a linear fit $T_{r}=a/\delta$ yielding $\left|a-2\pi \right|\leq 10^{-3}$, showing thus an excellent agreement with our prediction for the revival time.

The revivals correspond to a constructive quantum interference occurring at a time $T_{r}$ determined by the Hamiltonian (8) spectrum. Actually, this spectrum is only marginally affected by the coupling to the discrete state and has a nearly linear dependence of its eigenvalues $E_{n}≃n\hbar \delta$ (see the numerical analysis on Figure 3b). This result is valid for a wide range of energy gaps ℏδ. The condition (13) requires that for all values of *n*, $E_{n}T_{r}/\hbar =2\pi k_{n}$ with $k_{n}$ an integer. As $E_{n}≃n\hbar \delta$, we find $k_{n}=n$ and $T_{r}=2\pi /\delta$. Figure 4 confirms numerically the predictions of this simple revival model. We have plotted the revival time inferred from the exact resolution of the Schrödinger equations of the model with the Hamiltonian (8) as a function of 1/δ.

The above analysis provides a clear criterion for the suitability of the FQC for emulating irreversible dynamics. A necessary condition is the absence of revival during the considered time windows, i.e., $t_{f}<T_{r}$. This sets an upper bound on the energy gap, namely $\delta \leq \delta_{c}=2\pi /t_{f}$, or equivalently on the coupling strength $v\leq v_{c}=\hbar \sqrt{\Gamma /t_{f}}$, as both quantities are related by Equation (7). For the considered final time $t_{f}=10\;\Gamma$, we obtain the value $v_{c}=0.316\;\hbar \Gamma$ in very good agreement with the numerical results of Figure 3a. The region $v\geq v_{c}$ indeed corresponds to the onset of the gray zone, accounting for the degradation in the emulation of dissipative dynamics. In the next Section, we investigate the appropriate choice of the FQC model parameters in the different regimes of a driven two-level system.

## 4. Coupling of a Two-Level System to a Finite Discretized Continuum

**Figure 5:**
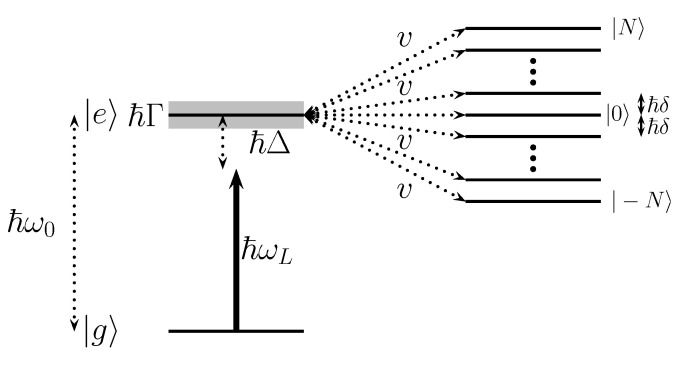
Two-level system driven by a laser pulse with a detuning (Δ), involving a stable ground state |g〉 and an excited state |e〉 coupled to a large but finite set of discrete levels. This coupling emulates an unstablity and yields an effective linewidth Γ for the transition.

*Model description and equations of motion*. In this Section, we consider a two-level atom with a stable ground state |g〉 and an unstable excited state |e〉 (see Figure 5), which is the standard model for spontaneous emission in quantum optics [23]. We denote by $\omega_{0}$ the transition frequency of this two-level system and assume that it is illuminated by a nearly-resonant laser of frequency $\omega_{L}≃\omega_{0}$. This external field drives the system with a Rabi coupling of frequency $\Omega_{0}$ between the two atomic levels. The excited state acquires a finite width Γ, due to its coupling with the continuum.

We now consider a $N_{\mathrm{tot}}=2N+3$-dimensional Hilbert space encapsulating the two-level quantum system and the FQC. Considering the driving term, the total Hamiltonian is given by

(14) $$H=\left(\begin{array}{ccccccc} 0 & \hbar \Omega_{0} & 0 & 0 & ... & 0 & 0\\ \hbar \Omega_{0} & \hbar \Delta & v & v & ... & v & v\\ 0 & v & -N\hbar \delta & 0 & 0 & ... & 0\\ 0 & v & 0 & -(N-1)\hbar \delta & 0 & ... & 0\\ 0 & . & . & 0 & . & 0 & ...\\ 0 & . & . & . & 0 & . & 0\\ 0 & v & 0 & 0 & ... & 0 & N\hbar \delta \end{array}\right).$$

on the basis $\{|g\rangle ,|e\rangle ,|\psi_{f}\rangle \}$ transformed in the rotating frame with the detuning $\Delta =\omega_{0}-\omega_{L}$. For a given dissipation rate Γ, the system is therefore determined by four independent driving ($\left\{\Omega_{0},\Delta \right\}$) and FQC ({N,δ}, or equivalently {N,v} from Equation (7)), parameters. We denote by |ψ〉 the quantum state of the full Hilbert space. The corresponding density matrix ρ=|ψ〉〈ψ| follows a unitary dynamics $i\hbar \frac{d\rho }{dt}=\left[H,\rho \right].$ We now focus on the non-unitary quantum dynamics in the reduced Hilbert space. Specifically, we consider the evolution of the 2×2 density matrix $\rho_{r}=P_{ge}\rho P_{ge}$, where $P_{ge}=\left|g\rangle \langle g\right|+\left|e\rangle \langle e\right|$ is the projector on the two-dimensional Hilbert space of the system. The reduced density matrix $\rho_{r}$ can be obtained by first solving the full unitary dynamics and then applying the projector. In order to highlight the role played by the FQC, the equation of motion for the reduced density matrix can be rewritten in the following form:

(15) $$i\hbar \frac{d\rho_{r}}{dt}=\left[H_{0},\rho_{r}\right]+S_{r}^{\mathrm{FQC}}.$$

The r.h-s contains the unitary driving of the system Hamiltonian $H_{0}=P_{ge}HP_{ge}$, as well as a source term accounting for the interaction with the FQC

(16) $$S_{r}^{\mathrm{FQC}}=\left(\begin{array}{cc} 0 & \lambda_{N}\\ \lambda_{N}^{*} & \eta_{N} \end{array}\right).$$

where $\lambda_{N}=v\sum_{i=2}^{2N+2}\rho_{gi}$ and $\eta_{N}=v\sum_{i=2}^{2N+2}\left(\rho_{ie}-\rho_{ei}\right)$. This source term drives effective non-unitary dynamics within the considered time interval and depends on the coherence between the FQC levels and the quantum system. The equations above contain no approximation and capture the full quantum dynamics of the two-level system coupled to a FQC.

*Non-Hermitian dynamics*. Here, we briefly review the equations of motion under an effective non-Hermitian Hamiltonian. Beyond their applications in nanophotonics, effective non-Hermitian Hamiltonians adequately describe the dynamics of open quantum systems in many experimental situations. For instance, this approach has been successfully used to explain the subradiance effects in large atomic clouds [28]. As in Section 2, the effective non-Hermitian Hamiltonian is obtained by deriving differential equations for the two-level system probability amplitudes $\left(c_{e},c_{g}\right)$. Using rotating wave-approximation, one finds $H_{\mathrm{eff}}=H_{0}+iH_{d}$ with $H_{0}=\hbar \Omega_{0}(|e\rangle \langle g|+h.c.)+\hbar \Delta |e\rangle \langle e|$ and $H_{d}=-\frac{\hbar }{2}\Gamma |e\rangle \langle e|$. The anti-Hermitian contribution $iH_{d}$ captures the decay towards the continuum. The evolution of the reduced density matrix under the influence of this effective Hamiltonian takes a form analogous to Equation (15)

(17) $$i\hbar \frac{d{\tilde{\rho }}_{r}}{dt}=\left[H_{0},{\tilde{\rho }}_{r}\right]+S_{r}^{\infty }$$

with a source term $S_{r}^{\infty }=i{\left[H_{d},{\tilde{\rho }}_{r}\right]}_{+}$ capturing the non-unitary dynamics (${\left[\;\right]}_{+}$ is an anti-commutator). Numerical analysis confirms that $S_{r}^{\infty }$ also corresponds to the limit of the FQC source terms $S_{r}^{\mathrm{FQC}}$ (16) within the large quasi-continuum limit N→+∞. At resonance (Δ=0), the Schrödinger equation in the presence of $H_{\mathrm{eff}}$ boils down to the equation of a damped harmonic oscillator for the probability amplitude $c_{e}$

(18) $${\ddot{c}}_{e}+\Gamma {\dot{c}}_{e}/2+\Omega_{0}^{2}c_{e}=0.$$

**Figure 6:**
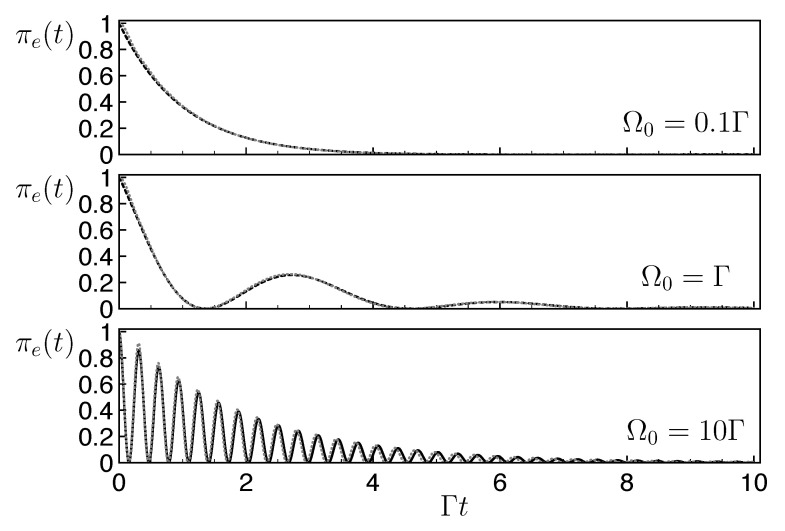
Non-Hermitian vs. FQC dynamics for the two-level system in the following regimes: over-damping ($\Omega_{0}=0.1\Gamma$, upper panel), critical ($\Omega_{0}=1\Gamma$, middle panel), and under-damping ($\Omega_{0}=0.1\Gamma$, lower panel) in the presence of an FQC with $N_{\mathrm{FQC}}=2N+1=61$ levels and v=0.3ℏΓ (full quantum dynamics, gray dotted line) or from the non-Hermitian dynamics (Equation (18), black dashed line). Both lines are superimposed, showing the excellent emulation of non-Hermitian dynamics with the considered FQCs.

One identifies the three usual dynamical over/critical/under-damping regimes determined by the ratio $\Omega_{0}/\Gamma$ (see the black dashed lines in Figure 6).

*Example of successful FQC-emulated dynamics*. In Figure 6, we investigate the suitability of a FQC with parameters {N,v}={30,0.3ℏΓ} for the emulation of non-Hermitian dynamics in these different regimes. We obtain the evolution of the excited state population $\pi_{e}\left(t\right)$ coupled to this FQC using a numerical resolution of the Schrödinger equation with the Hamiltonian (14), and compare it to the evolution under the non-Hermitian dynamics given by Equation (18). Excellent agreement is observed for the three distinct regimes, covering a wide range of $\Omega_{0}/\Gamma$ values. We investigate below how to determine the minimal number of levels of an adequate FQC.

*Quantitative mapping of successful FQCs for the emulation of two-level non-Hermitian dynamics*. Before proceeding to a more systematic analysis of the suitability of the FQC, we introduce a quantitative measure for the accuracy of FQC-emulated dynamics. Specifically, in the considered two-level system, we take the trace distance [29] between the reduced density matrices evolved respectively under the influence of a FQC (unitary evolution with *H* (14) followed by projection with $P_{eg}$) and following non-Hermitian dynamics (Equation (17)). This distance is defined for two density matrices ρ and σ by

(19) $$T\left(\rho ,\sigma \right)=\frac{1}{2}Tr\left(\sqrt{{\left(\rho -\sigma \right)}^{†}\left(\rho -\sigma \right)}\right).$$

In order to obtain a quantitative estimate of the fidelity over the whole considered interval, we use the mean trace distance over the considered time window:

(20) $$D_{2}\left(t_{f}\right)=\frac{1}{t_{f}}\int_{0}^{t_{f}}T\left(\rho_{e}\left(t\right),\sigma \left(t\right)\right)dt.$$

This definition in terms of trace distance coincides with the measure $D_{1}$ introduced in Equation (11) in the one-dimensional case.

**Figure 7:**
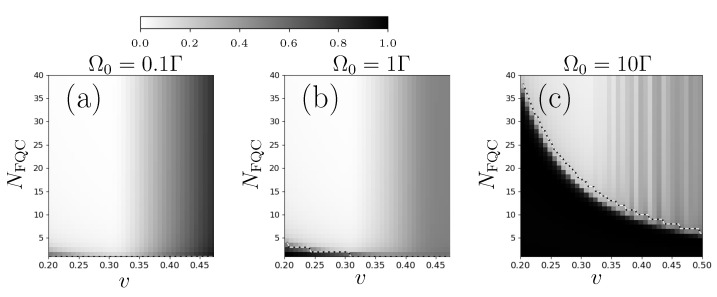
Quality of the emulation of non-Hermitian dynamics with a FQC: 2D plots of the mean trace distance (normalized to its maximum value) between the non-Hermitian model and the dynamics in a FQC model with parameters $N_{\mathrm{tot}}$, *v* (in units of ℏΓ) for the respective ratios $\Omega_{0}/\Gamma$ = 0.1 (**a**), 1 (**b**), 10 (**c**). The dotted black–white line corresponds to the number $N_{\max}\left(v\right)$.

As in Section 3, we proceed to a systematic study of the appropriate FQC parameters (N,v) for the emulation of non-Hermitian dynamics. We here separately consider the three different regimes evidenced by Equation (18) and we use the mean trace distance (20) between the respective density matrices evolving in the presence of a FQC ($\rho_{r}$) or following non-Hermitian dynamics (${\tilde{\rho }}_{r}$). The results are summarized in Figure 7a–c for the different ratios $\Omega_{0}/\Gamma$ corresponding to the three distinct regimes of non-Hermitian dynamics. In order to avoid the revival effect discussed in Section 3, we take a slightly shorter time interval $t_{f}=8\Gamma^{-1}$. A comparison between the mappings presented Figure 3a and Figure 7a–c reveals very different characteristics in the FQC emulation for the one- and two-level systems. For the one-level system, successful FQC emulation only requires the absence of revivals, associated with a condition $v\leq v_{c}$ independent of the FQC size *N*. Differently, we see for the two-level case that the number 2N+1 of FQC states has a critical influence on the fidelity of the FQC-emulated dynamics. These figures reveal an abrupt transition when the parameter *N* falls below a critical value N(v), depending on the coupling strength *v* for a given ratio $\Omega_{0}/\Gamma$. This raises the question of how to choose suitable FQC parameters.

*Suitability criteria for FQC*. Here, we determine the subset of FQC states that are significantly populated during the time evolution. Intuitively, this set should form the minimal FQC which accurately captures dissipative quantum dynamics. As can be seen below, the populated modes essentially depend on the Rabi frequency $\Omega_{0}$ and dissipation rate Γ.

This situation is reminiscent of the dynamical Casimir effect (DCE), in which a continuum of vacuum electromagnetic modes becomes gradually populated under the harmonic motion of a moving mirror (See Ref. [30] for a review). In the DCE, the mirror oscillation at a frequency $\Omega_{m}$ induces the emission of photons of frequencies $\omega \leq \Omega_{m}$ in initially unpopulated electromagnetic modes. A similar effect is observed with a moving two-level atom [31,32] in the vacuum field. We find below that our FQC model with a Rabi driving reproduces these features, with the emergence of sidebands at the Rabi frequency in the FQC population. As in the DCE, the external drive provides energy to the system, which eventually leaks into the continuum.

To analyze this effect, we introduce the expansion

(21) $$|\psi \left(t\right)\rangle =c_{e}\left(t\right)|e\rangle +c_{g}\left(t\right)|g\rangle +\sum_{p=-N}^{N}c_{p}\left(t\right)|p\rangle$$

into the Schrödinger equation. A projection on the kth state of the FQC yields a differential equation for the coefficient $c_{k}\left(t\right)$ driven by the excited state probability amplitude $c_{e}\left(t\right).$ This equation is formally solved as

(22) $$c_{k}\left(t\right)=-\frac{iv}{\hbar }\int_{0}^{t}c_{e}\left(t^{\prime }\right)e^{ik\delta t^{\prime }}dt^{\prime }.$$

**Figure 8:**
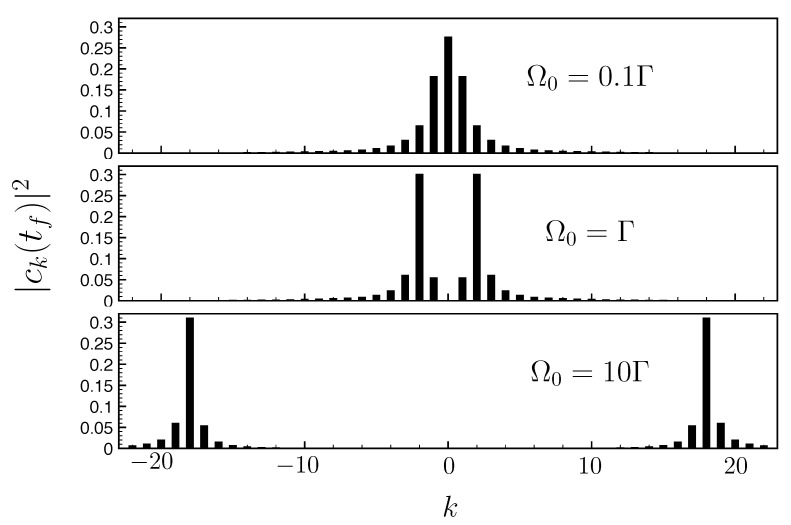
Distribution of populations: Occupation probability $|c_{k}\left(t_{f}\right){|}^{2}$ at the final time $t_{f}$ as a function of the level *k* of the FQC for $\Omega_{0}=0.1\;\Gamma$ (lower panel), $\Omega_{0}=\Gamma$ (middle panel) and $\Omega_{0}=10\;\Gamma$ (upper panel). Parameters: 2N+1=45 levels, and with v=0.3ℏΓ.

In the long-time limit, the coefficient $c_{k}\left(t\right)$ tends towards the Laplace transform of the excited state amplitude at the frequency kδ (up to a constant factor). In order to estimate the occupation probability $|c_{k}{\left(t\right)|}^{2}$ at time $t<t_{f}$, we use the probability amplitude ${\tilde{c}}_{e}\left(t\right)$ given by non-Hermitian dynamics (Equation (18)). The latter is indeed an excellent approximation of the excited state probability $c_{e}\left(t\right)$ in coupling to a sufficiently large FQC (see Figure 6). We find

(23) $$c_{k}\left(\infty \right)=\frac{v\Omega_{0}}{\hbar \sqrt{\Delta_{0}}}\left[\frac{-1}{-\frac{\Gamma }{4}+\frac{i\sqrt{\Delta_{0}}}{2}+ik\delta }+\frac{1}{-\frac{\Gamma }{4}-\frac{i\sqrt{\Delta_{0}}}{2}+ik\delta }\right]$$

with $\Delta_{0}=4\Omega_{0}-\frac{\Gamma^{2}}{4}$. Figure 8 shows the probability occupations $|c_{k}\left(t_{f}\right){|}^{2}≃{\left|c_{k}\left(\infty \right)\right|}^{2}$. These distributions exhibit two sidebands centered about *k* values, such that $|k|\delta ≃\Omega_{0}$, symmetrically distributed around k=0 for our choice of Δ=0. A similar generation of sidebands is observed for the dynamical Casimir effect [30]. These occupancy probabilities actually determine the number of relevant FQC states and the size of the minimal appropriate FQC. Indeed, we have indicated in Figure 7a–c the maximum occupancy number $N_{\max}\left(v\right)$ as a function of the coupling strength *v*. This quantity is defined as $|c_{N_{\max}\left(v\right)}\left(t_{f}\right){|}^{2}=\max_{n}\left\{\right|c_{n}\left(t_{f}\right){|}^{2}\}$ for the considered coupling strength *v* and Rabi frequency $\Omega_{0}$. As the occupation peak approximately corresponds to the Rabi frequency, we expect $N_{\max}\left(v\right)≃\Omega_{0}/\delta =\Omega_{0}\hbar^{2}\Gamma /\left(2\pi v^{2}\right)$ from Equation (7). In Figure 7a–c, the line representing the maximum occupancy number $N_{\max}\left(v\right)$ is almost superimposed on the interface between the suitable and unsuitable FQCs (white/grey zones, respectively). This confirms that the suitable FQCs are those that host all the significantly populated levels. The population of each Fourier components is represented for different $\Omega_{0}/\Gamma$ ratios in Figure 8: in the weak coupling limit $\Omega_{0}\ll \Gamma$, $N_{\max}\left(v\right)$ is mainly determined by the dissipation rate Γ, while in the strong coupling limit $\Omega_{0}\gg \Gamma$, it scales linearly with the Rabi frequency $\Omega_{0}$ (for v=0.3, $N_{\max}\left(v\right)≃1.77\Omega_{0}$).

## 5. Non-Markovian Dynamics and Adaptive Quasi-Continuum

In this Section, we analyze quantitatively the finiteness-related effects in the evolution of an FQC-coupled quantum system. First, we establish the connection between the presence of revivals (discussed in Section 3) and a measure of non-Markovianity applied to the FQC-emulated dynamics. Second, we show that the FQC structure of equidistant energy levels induces a mismatch of the effective Rabi frequency and decay rates when compared to the equivalent parameters in the non-Hermitian model. Solving this issue suggests an adaptative structure of FQCs, discussed below, capable of reproducing non-Hermitian dynamics with a considerably reduced number of states.

### 5.1. Revivals and Non-Markovianity

Revivals in the excited state probability $\pi_{e}\left(t\right)$, discussed in Section 3 for the single-level system, also occur in the two-level FQC emulated dynamics for large values of coupling strength *v*. Such revivals are indeed a symptom of non-Markovian dynamics in the FQC; their exact form depends on the initial quantum state and therefore reveals a memory effect in the quantum evolution. Despite the successful emulation of dissipative dynamics over a given time interval, these revivals show that some information about the initial state has been transmitted and stored in the FQC. The revival appears as a kind of constructive interference effect when information about the initial state, stored in the FQC, returns to the system. Non-Hermitian dynamics (17) are Markovian, and so the emergence of non-Markovianity reveals a discrepancy between the FQC-emulated system and the ideal irreversible case.

These considerations suggest quantitatively studying the non-Markovianity of the FQC-emulated dynamics. We proceed by using the measure from Ref. [33], summarized below for convenience. This measure uses the trace distance T(ρ,σ) (19), which has a direct interpretation in terms of the distinguishability of the associated quantum states. Indeed, if we consider an emitter which randomly prepares one of the two quantum states {ρ,σ} with equal probability, the probability of an observer successfully identifying the correct quantum state through a measurement is simply $\frac{1}{2}\left(1+T\left(\rho ,\sigma \right)\right)$. Markovian processes correspond to a decreasing trace distance for any set of states following the quantum evolution associated with the process. In this case, no information likely to improve the dinstinguability of the states {ρ(t),σ(t)} is acquired by the system during the evolution. The unitary evolution operator of a closed quantum system, and more generally complete positive trace-preserving maps, fall into this category. Conversely, non-Markovian quantum processes are those that exhibit at least a temporary positive variation of the trace distance for some pair of initial states. This increase witnesses a flow of information from the environment back to the system.

To obtain a quantitative measure, one introduces the rate of variation of the trace distance for a given quantum process

(24) $$\sigma_{\rho_{1}^{0},\rho_{2}^{0}}\left(t\right)=\frac{d}{dt}T\left(\rho_{1}\left(t\right),\rho_{2}\left(t\right)\right).$$

where $\rho_{1,2}\left(t\right)$ are two density matrices undergoing the quantum process under consideration and therefore following the same evolution operator/dynamic equation, but with distinct initial conditions $\rho_{1,2\;}\left(0\right)=\rho_{1,2}^{0}$. Quantum processes with $\sigma_{\rho_{1}^{0},\rho_{2}^{0}}\left(t\right)>0$ correspond to an increasing trace distance, and therefore a flow of information from the environment to the system. The non-Markovianity measure is given by [33]

(25) $$\Sigma \left(t\right)=\max_{\rho_{1}^{0},\rho_{2}^{0}}\int_{0}^{t}dt^{\prime }\;\Theta \left(\sigma_{\rho_{1}^{0},\rho_{2}^{0}}\left(t^{\prime }\right)\right)\;\sigma_{\rho_{1}^{0},\rho_{2}^{0}}\left(t^{\prime }\right)$$

**Figure 9:**
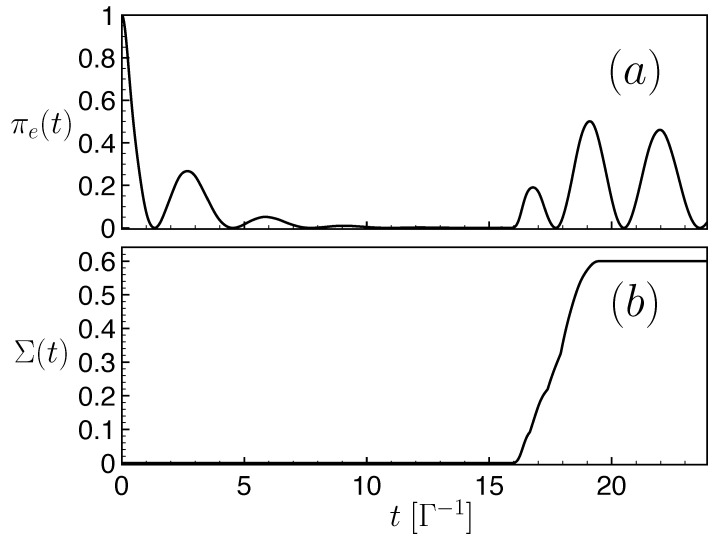
Revivals and non-Markovianity: (**a**) Time evolution of the population in the unstable state $\pi_{e}\left(t\right)$. (**b**) Measure of the non-markovianity Σ(t) (see text) of a two-level system composed of 2N+3=53 levels as a function of time. Parameters: $\Omega_{0}=1\;\Gamma$, v=0.25ℏΓ. The non-Markovianity measure is estimated within a 1% accuracy by sampling over a set of 256 initial states.

The Heaviside function Θ(x) (s.t. Θ(x)=1 if x≥0 and Θ(x)=0 for x<0) guarantees that only time intervals with an increasing trace distance effectively contribute to the integral. The quantity Σ(t) is obtained by considering all possible initial quantum states $\rho_{i}^{0}=|\psi_{i}\rangle \langle \psi_{i}|$ (with $|\psi_{i}\rangle$ a generic quantum state of the full Hilbert space), and the considered evolution corresponds to $\rho \left(t\right)=P_{\mathrm{eg}}e^{-iHt/\hbar }\rho^{0}e^{iHt/\hbar }P_{\mathrm{eg}}$, where *H* is the Hamiltonian (14) and $P_{\mathrm{eg}}$ the projection operator introduced earlier for the two-dimensional subspace. In Figure 9b, we have plotted the evolution of the non-Markovianity Σ(t) as a function of time for a given FQC, to be compared with the time evolution of the excited-state population $\pi_{e}\left(t\right)$ in Figure 9a. We deliberately chose a time window during which a revival was observed. Figure 9a,b reveal that a sharp increase in the non-Markovianity Σ(t) occurs at the onset of the probability revival. The non-Makovianity Σ(t) thus provides another determination of the time window over which the FQC dynamics accurately emulate an irreversible non-Hermitian evolution.

### 5.2. Adaptive FQC

The study carried out in Section 4 reveals the minimal size of suitable FQCs scales with the Rabi frequency $\Omega_{0}$. Here, we go one step further and propose adapting the FQC’s structure depending on the Rabi coupling $\Omega_{0}$. We no longer consider exclusively flat FQCs with equidistant levels around the excited state energy. Instead, we study adaptive FQCs with an enhanced density of states around the occupation peaks depicted in Figure 8. As seen below, such adaptive FQCs yield an optimized emulation of non-Hermitian dynamics.

**Figure 10:**
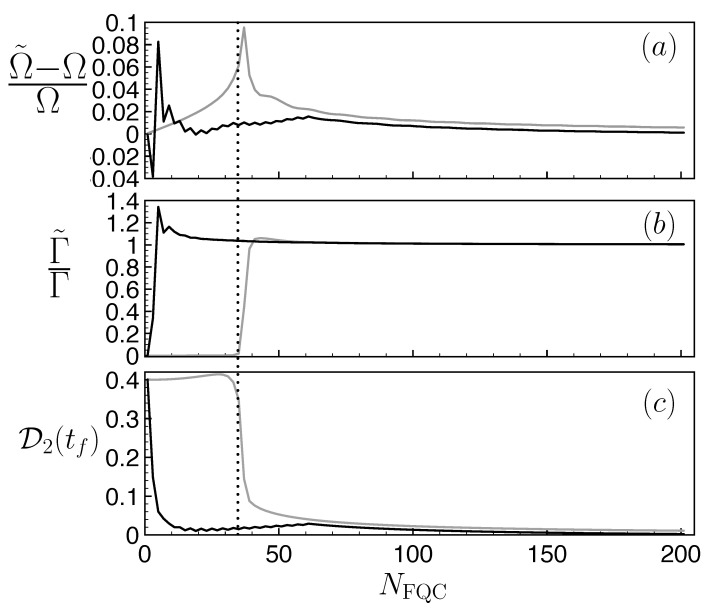
Mismatch between the effective Rabi frequency (**a**), damping rates (**b**), and mean trace distance (**c**) as a function of the size $N_{\mathrm{FQC}}$ obtained by comparing the FQC model with the non-Hamiltonian model. We have plotted the effective parameters obtained from a flat FQC made of equidistant levels (gray solid line) and for an FQC with an adaptative structure with removed central states (black solid line). The dotted line corresponds to $N_{\mathrm{FQC}}=35$, considered in Figure 11. Parameter v=0.3ℏΓ.

We begin by investigating the influence of the discrete FQC structure on the emulated quantum dynamics. Figure 7c exhibits a slightly gray zone associated with a slight mismatch between the FQC evolution and the non-Hermitian dynamics. This is the regime we wish to investigate. For this purpose, we consider non-Hermitian dynamics (18) in the strong coupling regime $\Omega_{0}\gg \Gamma$. The corresponding excited-state population reads

(26) $$\pi_{e}\left(t\right)=e^{-\Gamma t/2}\cos^{2}\left(\Omega t\right),$$

with $\Omega =\Omega_{0}{\left(1-{\left(\Gamma /4\Omega_{0}\right)}^{2}\right)}^{1/2}$. An effective Rabi frequency $\left(\tilde{\Omega }\right)$ and dissipation rate $\left(\tilde{\Gamma }\right)$ for the FQC dynamics are obtained by fitting the excited-state population ${\tilde{\pi }}_{e}\left(t\right)$ with the form (26) of exact non-Hermitian dynamics. The corresponding results are represented as a solid gray line in Figure 10a,b for different FQCs.

The discrepancy between the FQC model and the ideal non-Hermitian case can be explained through a closer examination of the integration Kernel (2), or more precisely its equivalent for the two-level case. To reproduce non-Hermitian dynamics, the integration Kernel must take a form analogous to Equation (4). In this case, the frequency mismatch $\Delta \Omega =\tilde{\Omega }-\Omega$ cannot be attributed to a Lamb shift effect, as the principal part of the kernel in Equation (6) cancels out in the presence of a symmetric FQC with a homogeneous coupling constant. The slight frequency shift is therefore a signature of the non-Markovianity of the FQC dynamics, i.e., of the residual error committed by replacing Equation (2) with Equation (4). The corresponding approximations, namely of short kernel memory and the extension of the integral in Equation (4) to infinity, are indeed jeopardized by the discrete FQC structure. Intuitively, the discrete states of zero-energy (i.e., of energy close to the unstable excited state) can increase the error. We also note that these central states are not significantly populated in the FQC dynamics: the highly populated levels correspond to peak population sidebands centered on $\pm \Omega_{0}/\delta$.

These observations raise the question of the relevant optimal FQC structure in this regime. From Figure 3b, the central FQC state eigenenergies undergo the largest shift from the linear dispersion relation expected from an ideal continuum. Furthermore, Figure 8c shows that, in the strong driving regime ($\Omega_{0}\gg \Gamma )$, the final population of these states is very small. These considerations suggest that the central components of the FQC play a minor role, or even a deleterious role.

To confirm this intuition, we studied a different FQC model obtained from the former FQC, by removing the states close to the E=0 energy while preserving the symmetry of the distribution. The corresponding results are shown in Figure 10a–c (solid black line). For large FQC sizes, both the flat and adaptive FQC provide a good emulation of non-Hermitian dynamics, although the latter had the same error in the $\tilde{\Omega },\tilde{\Gamma },D_{2}$ parameters with a much smaller size. For small sizes $N_{\mathrm{FQC}}\leq 30$, the spectrum of the regular flat FQC is too narrow to include the highly populated Rabi sidebands of Figure 8c. Consequently, small regular FQCs produce a negligible effective damping rate $\tilde{\Gamma }$. On the other hand, by construction, the adaptive FQC contains states nearby these sidebands. Thus, even small adaptive FQCs $N_{\mathrm{FQC}}≃10$ already give an effective damping rate $\tilde{\Gamma }$ close to the appropriate value. At intermediate sizes ($10\leq N_{\mathrm{FQC}}\leq 50$), adaptive FQCs also outperform regular FQCs: a strong improvement is observed in the agreement between the effective Rabi frequency $\tilde{\Omega }$ and damping rate $\tilde{\Gamma }$ with their non-Hermitian counterparts Ω,Γ, as well as a significant reduction in the mean trace distance compared to exact non-Hermitian dynamics. We conclude that, for the damped Rabi dynamics considered, adaptive FQCs with a tailored distribution (involving mostly states close to the Rabi frequency sidebands $\pm \Omega_{0}/\delta$ and presenting a hole in the central zone near the unstable state energy (E=0)) provide a higher fidelity to non-Hermitian dynamics with constant resources, i.e., with the same number of states and for an identical time window.

**Figure 11:**
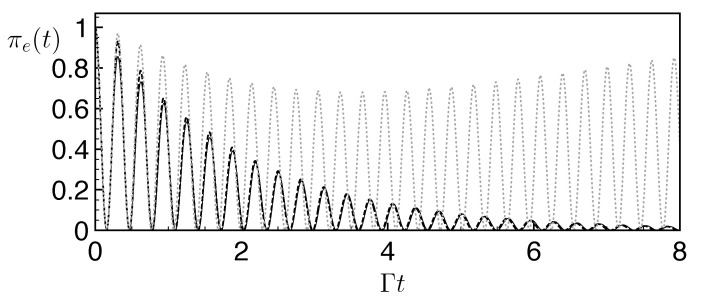
FQC-emulated dynamics in the strong coupling regime ($\Omega_{0}=10\Gamma$): excited population as a function of time for a flat FQC (dotted gray line) made of $N_{\mathrm{FQC}}=2N+1=35$ equidistant levels and for an adaptative FQC (dashed black line) made of $N_{\mathrm{FQC}}=2N=34$ levels. For both FQCs, we used the parameters v=0.3ℏΓ. The solid gray line represent the exact evolution expected from the non-Hermitian dynamics (Equation (26)), and is almost superimposed onto the adaptative FQC results.

Figure 11 provides an example, where both kinds of FQC (flat vs. adaptive) produce very different qualitative behaviors, while having a very similar number of states. While the coupling to a regular equidistant FQC cannot account for the damping of the Rabi oscillation, the quantum system coupled to the adaptive FQCs yields an excellent agreement with the predicted non-Hermitian dynamics (Equation (26)).

## 6. Conclusions

In conclusion, we discussed the emulation of non-Hermitian quantum dynamics during a given time window with a finite quasi-continuum composed of discrete states. We specifically considered the exponential decay of an unstable state, and the Rabi driving of a two-level quantum system exhibiting an unstable state. We characterized the short- and long-time deviations of the FQC-emulated system compared to the exact non-Hermitian case. Short-time deviations can be interpreted in terms of the Zeno effect, while the long-term deviations correspond to a probability revival that can be quantified using a measure of non-Markovianity. We provided a criterion for the adequacy of the discrete FQCs considered by evaluating the occupancy probabilities of the quasi-continuum states. There is a trade-off between using FQCs involving a large number of states and achieving high accuracy in emulating non-Hermitian dynamics. We showed that, in the strong coupling regime, this trade-off can be significantly improved by considering FQCs with an adapted density of states. This study is potentially relevant for many body systems, where a given subsystem can be coupled to a large set of states corresponding to the surrounding bodies [7]. Quantum dots coupled to nano-wires are a promising platform for implementing low-dimensional systems coupled to FQCs [34,35]. This work also paves the way for the emulation of non-Hermitian dynamics with a finite set of states. A long-term goal is to integrate a tunable dissipation within quantum simulators [7]. Different methods have been investigated to reach this goal, relying on the Zeno effect [36,37,38,39], atom losses [40,41], and multichromatic Floquet [42], to name a few.

## Data Availability

Data is contained within the article or supplementary material.
